# A Review of Patient Bed Sensors for Monitoring of Vital Signs

**DOI:** 10.3390/s24154767

**Published:** 2024-07-23

**Authors:** Michaela Recmanik, Radek Martinek, Jan Nedoma, Rene Jaros, Mariusz Pelc, Radovan Hajovsky, Jan Velicka, Martin Pies, Marta Sevcakova, Aleksandra Kawala-Sterniuk

**Affiliations:** 1 Department of Cybernetics and Biomedical Engineering, Faculty of Electrical Engineering and Computer Science, VSB-Technical University of Ostrava, 17. Listopadu 2172/15, 708 00 Ostrava-Poruba, Czech Republic; michaela.sidikova@vsb.cz (M.R.); radovan.hajovsky@vsb.cz (R.H.); jan.velicka@vsb.cz (J.V.); martin.pies@vsb.cz (M.P.); marta.sevcakova@vsb.cz (M.S.); 2 Department of Telecommunications, Faculty of Electrical Engineering and Computer Science, VSB-Technical University of Ostrava, 17. Listopadu 2172/15, 708 00 Ostrava-Poruba, Czech Republic; jan.nedoma@vsb.cz; 3 Institute of Computer Science, University of Opole, ul. Oleska 48, 45-052 Opole, Poland; m.pelc@greenwich.ac.uk; 4 School of Computing and Mathematical Sciences, Old Royal Naval College, University of Greenwich, Park Row, London SE10 9LS, UK; 5 Faculty of Electrical Engineering, Automatic Control and Informatics, Opole University of Technology, ul. Proszkowska 76, 45-758 Opole, Poland

**Keywords:** sensors, vital sign monitoring, digital signal processing, biosignals

## Abstract

The analysis of biomedical signals is a very challenging task. This review paper is focused on the presentation of various methods where biomedical data, in particular vital signs, could be monitored using sensors mounted to beds. The presented methods to monitor vital signs include those combined with optical fibers, camera systems, pressure sensors, or other sensors, which may provide more efficient patient bed monitoring results. This work also covers the aspects of interference occurrence in the above-mentioned signals and sleep quality monitoring, which play a very important role in the analysis of biomedical signals and the choice of appropriate signal-processing methods. The provided information will help various researchers to understand the importance of vital sign monitoring and will be a thorough and up-to-date summary of these methods. It will also be a foundation for further enhancement of these methods.

## 1. Introduction

The average life expectancy of the population is gradually increasing, so vital sign monitoring in aging populations is becoming of utmost importance [1]. The main reason for writing this review paper was the need for a thorough review covering continuous monitoring of various vital parameters obtained from sensors mounted to beds. This is important, as vital parameters provide valuable information about the patient’s condition and allow for a timely response in case of an emergency [2,3]. They can also be successfully applied in various tele-medical solutions. As society is rapidly aging, emphasis is being placed on the requirements for the monitoring of seniors both in hospitals and in home care-based units [4]. In hospitals, it is possible to continuously monitor the key parameters, providing valuable information regarding patients’ conditions and alerts in case of potential emergencies [2].

Recent developments in communication technologies now allow for patient data, including routine vital signs, to be examined remotely. Remote monitoring, or telemonitoring, uses electronic and telecommunication technologies to deliver and support health care from a distance. Depending on the setting and purpose, the patients and the system that examines, analyzes, and interprets the data may be a few meters apart, for example, or located in a different room or city. Current telemonitoring challenges include the lack of a range of suitable sensors, the large weight and size of the entire system or its components, battery life, available bandwidth, network coverage, and the cost of transmitting data over public networks. Telemonitoring also has the ability to produce a wealth of data—but this requires interpretation to make it clinically useful [1,5,6].

Current medical applications of wireless sensor networks aim to monitor heart problems, breathing/pressure problems, etc. Currently, integrating existing medical technology with wireless networks in specialized areas is a more common topic [7]. Small wearable non-intrusive sensors [8] will facilitate automated large data collection. These sensors will reduce regular visits to clinics and thus reduce medical facility expenses. Future research work will benefit the entire field of health [9]. Smart vests or smart t-shirts [10,11,12,13] are also in development as necessary systems that can be worn for monitoring vital signs. Various sensors can be integrated into the wearable device while collecting biological signals in an unobtrusive and non-invasive manner.

In order to enable continuous monitoring without restricting patient movement or causing discomfort, a sensor system can be mounted just underneath the entire mattress [2,3]. Research regarding contactless monitoring of vital signs has been recently expanded with the use of conventional video cameras (for monitoring purposes) [14]. Most of the current work in this field has been performed over short periods (usually up to several minutes) under strictly controlled conditions with relatively healthy volunteers [15].

For non-contact physiological assessments, computer vision-based methods appear to be a favorable approach that could be reliable, safe, cost-effective, and suitable for remote and long-term monitoring. In addition, video technology allows for measurements of multiple individuals occasionally and simultaneously in groups [14,16].

Watanabe et al. [17] developed a non-invasive system based on a pneumatic system that can be used to measure the heart rhythm, breathing, snoring, and body movements of a subject in bed. A thin air-sealed cushion is placed under the mattress of the subject’s bed, and small movements attributable to automatic vital human functions are measured as pressure changes using a pressure sensor with an almost flat frequency response from 0.1 to 5 kHz and a sensitivity of 56 mV/Pa. Using the newly developed system, heart rhythm, respiration, apnea, snoring, and body movements can be measured. In addition, the optimal signal-to-noise ratio is given to evaluate the reliability of the heart rate measurements. Heart rates were measured for four different body positions, thirteen different subjects, four different bed mattresses, and three different sensor positions.

Mahdavi and Rosell-Ferrer [18] focused on a system that is based on a magnetic induction sensing method designed to derive the presence of a person in the bed, respiration, and cardiac activity. It consists of two coils for excitation and detection. The new detection coil is based on a concentric planar gradiometer to cancel the primary field. The signal acquisition system was designed using simple electronics to avoid complexity and an expensive system.

In 2014, Waltisberg et al. [19] introduced a new approach to the evaluation of smart bed systems that would enable continuous vital sign measurements. They demonstrated that estimation accuracy (or error) and measurement coverage time are key performance metrics, describing performance trade-offs in practical smart bed systems. Based on a typical smart bed system that uses a force transducer array located between the bed mattress and the frame, it evaluates the effects of various signal filtering options to illustrate viable design options using our accuracy and coverage analysis. In a full-day recording study, six participants were observed, and their respiratory rate was estimated. Measurement coverage is a basic metric that should be analyzed along with the accuracy in evaluating the performance of smart bed systems.

Self-experimentation allows people to explore what behavioral changes lead to better health. These experiments are very challenging to conduct in a scientifically validated way while adapting to the realities of everyday life. Daskalova et al. [20] presented a set of design principles for guided self-experiments that aimed to reduce the barriers to self-experimentation. They demonstrated the values by implementing them in SleepBandits, an integrated system that includes a smartphone app for sleep experiments. The SleepBandits app guides the user through the experiment, automatically collects data from embedded sensors and user inputs, and calculates and presents the results in real time.

One of the main examples is a non-invasive continuous monitoring of blood pressure pulse wave speed based on the pulse arrival time (PAT) derived from ECG and PPG signals, which is a very promising parameter [21,22]. One example of how to subtly obtain a PAT was presented by Baek et al. in 2012 (see: [23]) when they integrated cECG and rPPG technologies into a chair. In addition, BCG has been integrated to increase the robustness of the heart rate measurements between rhythms.

Vehkaoja et al. [24] designed an inconspicuously integrated bed system for monitoring physiological parameters during sleep. The system uses textile electrodes integrated with a sheet to measure multiple channels of the electrocardiogram, which are selected one-by-one to detect the RR interval. A force transducer is also located under the bed to detect breathing and movement. Motion information has also been used to facilitate heart rate detection.

Yu et al. [2] introduced a new unobtrusive multi-modal sensor for the purpose of physiological parameter monitoring, including cECG, rPPG, and magnetic induction (MI) monitoring in one sensor. The proposed sensor system includes sensor nodes that are designed and optimized for integration into a grid array of multiple sensors in a bed and a central control cabinet for data acquisition and processing. Therefore, it has very universal use and is suitable for discreet monitoring of vital functions, both in a professional environment and in a home care environment.

Based on the literature search, several research gaps emerged, which will be addressed in the following chapters of this study. The main contributions and purpose of this study include:

- An overview of various vital signs obtained from sensors placed on the beds.
- An overview of possible sensors that can be used for monitoring.
- Potential applications of small wearable non-intrusive sensors.
- Possibilities and challenges of vital sign remote monitoring.

The rest of this article is organized as follows: Section 2 will describe the vital sings that could be measured during patient bed monitoring. Section 3 will describe the sensors that could be used for monitoring. Next, Section 4 deals with sleep quality monitoring. Vital sign monitoring challenges will be included in Section 5. The discussion will be presented in Section 6, and finally, Section 7 will contain a proposal for future work and conclusions.

Table 1 summarizes the links to the subsections describing the most important sensors used in this review for selected vital functions.

## 2. Vital Signs

Measurements of the vital signs include the most important parameters, such as [25,26]:

- heart rate,
- blood pressure,
- arterial oxygen saturation,
- respiratory rate.

In clinical practice, these can be mainly in intensive care units (ICU), as they can be monitored by the following devices [27,28]:

- electrocardiogram (ECG),
- photoplethysmogram (PPG),
- arterial blood pressure sensors (invasive) or blood pressure cuffs (non-invasive).

It is possible to distinguish between various vital signs, such as those listed below:

- capacitive ECG (cECG) [2,29],
- reflective PPG (rPPG) [30,31],
- magnetic induction monitoring (MR) [32],
- ballistocardiography (BCG) [33],
- camera-based methods:
  – photoplethysmographic imaging [34,35],
  – infrared thermography [36,37] (one of the most popular inconspicuous monitoring methods).

In order to facilitate discreet (non-invasive) monitoring of various physiological parameters, the following techniques have been integrated into everyday objects, such as:

- beds (cECG [38,39,40], MR [18], and a BCG [41]);
- car seats (cECG [42] and MR [43]),
- wearable devices (cECG [44], rPPG [45,46,47,48] a MR [49]).

**Figure 1:**
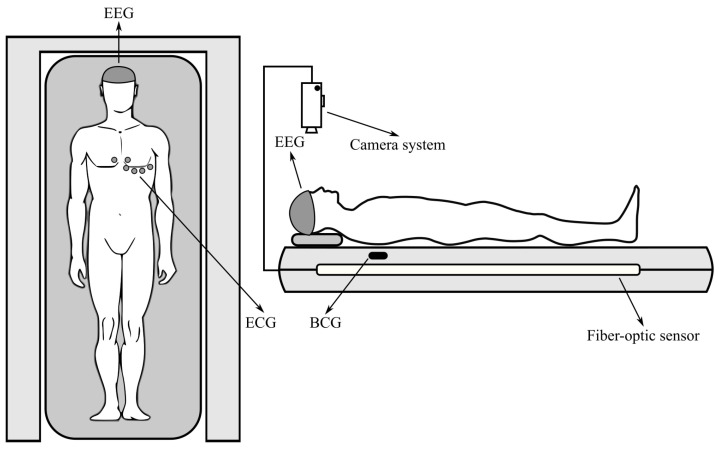
Location of the measuring systems.

Figure 1 illustrates the layout of all measurement systems that have been applied for vital sign monitoring and are presented in this work.

### Ballistocardiography

Measurement and processing of BCG signals for vital sign monitoring is very often used. Brüser et al. [50] described both sensor and signal processing improvements for discreet, long-term heart (and respiratory) rhythm monitoring using only vibrations obtained from non-invasive sensors. They described a system of inconspicuous monitoring of vital functions using a series of new BCG optical sensors located under the mattress. They further analyzed the spatial sensitivity of the systems and compared their concept with a more conventional BCG system based on a single electromechanical film (EMFi) sensor. Their results suggested that the proposed multi-channel optical system could be potentially useful in reducing errors in heart rate estimation among rhythms and would also enable the analysis of more complex respiratory patterns.

Another solution involving sensors (Emfit multi-channel pressure-sensing film) integrated in mattresses was proposed by Kortelainen et al. in [51], which enabled the measurement of BCG signals during sleep. The heart rate was calculated using the cepstrum method using the fast Fourier transform (FFT) for short-term windows, including a pair of subsequent heartbeats. The FFT scatter was reduced by averaging multi-channel data in the frequency domain.

Inconspicuous inter-bit interval (IBI) detection from BCG is useful for monitoring cardiac activity from home. It enables the calculation of heart rate variability (HRV), which is a crucial indicator for heart condition assessment. Most studies apply single sensor-based systems. For example, the authors implemented and used a built-in sensor system in order to improve measurement coverage and IBI estimation accuracy, and they also proposed a mode-switching algorithm that would solve the problem in the field of signal selection and multi-channel data fusion using a linear regression model and Kalman filter. In addition, they designed a peak detection algorithm to estimate the IBI from each channel signal. The algorithm was verified by approximately 48-hour BCG recordings of 24 subjects with different sleep positions [52].

Xie et al. [53] introduced an inconspicuous BCG-based heart rate (HR) monitoring system using a piezoelectric sensor that can be built into a chair or bed. It works in a way where it converts small body vibrations caused by a heartbeat into a BCG signal, and a new algorithm is applied for HR estimation based on the BCG signals. The proposed algorithm uses the Hilbert transform to reveal the frequency content of the J-peaks in the BCG signal for HR estimation. Viterbi decoding was used to improve the accuracy of HR for a noisy BCG signal by finding the most probable path of the time–frequency plane of state and space.

Another interesting solution includes multiple-instance vocabulary learning (DL-FUMI), which is used in order to estimate heart rate among rhythms and to characterize heart rate signatures from BCG signals recorded by a hydraulic bed sensor. DL-FUMI estimates the “concept of heart rhythm”, which is a personal model of the heart rhythm of a personal BCG. The proposed DL-FUMI formulates heart rate detection and heart rate characterization as a multi-instance learning issue to address the uncertainty associated with aligning BCG signals with the fundamental truth during training. This was preseted in work by Jiao et al. [54], where the authors showed that the estimated heartbeat concept obtained by DL-FUMI is an effective heartbeat prototype and achieves excellent performance over comparison algorithms.

It is also possible to mention the multi-instance learning method (MIL), extended by the multi-instance function (eFUMI), which is applied to BCG signals produced by a hydraulic bed sensor. This work [55] aims to learn the concept of the “personal heartbeat” of individuals. This heartbeat concept is a prototype (or “signature”) that characterizes the heartbeat pattern of individuals in BCG data. The eFUMI method models the problem of learning the concept of the presence signal from the BCG signal as a MIL problem. This approach elegantly addresses the uncertainty associated with the BCG signal. Due to the BCG training signal associated with the ground truth signal, training “bags” can be labeled only with binary labels that indicate whether the training bag contains a heart rate signal or not. Then, with the help of these eFUMI bags, they will learn a customized heart rhythm concept for the subject as well as several heartless background concepts. After becoming acquainted with the concept of heartbeat, heart rate detection, and heart rate estimation, it can be used for the tested data.

## 3. Applied Sensors

The most important part of any wearable device is the sensor, as it allows us to collect data and then analyze it. By improving technology, we can sense many different parameters. For the purpose of appropriate measurements, various sensors are being applied [8].

### 3.1. Fiber Bragg Grating

One of the most frequently used types of fiber sensors is grid sensors, which can be applied for the purpose of monitoring vital functions of the human body, including heart rate and respiratory activity [56].

In [57], the authors presented a system that enabled the successful detection of life-threatening situations of patients tied directly to the hospital bed (patients unable to move or those with minimal movement capabilities). The main aim of this work was to create a fiber Bragg grating (FBG)-based sensor that did not need to be mounted directly to the patient’s body.

Ref. [58] had a similar aim to the one mentioned above, and the result of their study was a sensor characterized by a technology applied for detecting respiratory activity with an error rate of less than 1%.

Similarly good results were also presented in [59,60,61], where sensory systems designed to detect both heart rate and respiratory rate were described. These papers also proved that their developed measuring systems provided comparable results to those obtained from conventional breath and pulse detection devices.

A very interesting paper [62] introduced the project of a pad placed under the patient’s body. The pad was made of a thin sheet of plexiglass and Bragg grid sensors attached with an epoxy glue. The results obtained were very good, and MRI testing was also performed.

References [63,64] also described the use of Bragg grid sensors, but they focused only on heart rate detection. The authors described a sensor built directly into the mattress, where the error rate of the sensor was less than 1 beat per minute.

Table 2 shows a broad comparison of the most widely used approaches applied to FBG sensors.

### 3.2. Interferometers

The second group of optical fiber sensors are interferometric sensors, which can be characterized by a very high sensitivity to the measured quantities. Fiber-optic interferometric sensors in biomedical applications can be divided into the four basic categories listed below:

- Fabry–Perot interferometer
- Mach–Zehnder interferometer
- Michelson interferometer
- Sagnac interferometer

In [65], the authors described the implementation of a measuring spiral fiber mounted to a mattress. Their research aimed to monitor the development of heart rhythms and respiratory activity. The results of the study were based on experimental measurements of 14 subjects, showing a 98.18±1.40% sensitivity and 97.04±4.95% precision for heart rate measurements and a 90.06±7.49% sensitivity and 94.21±3.70% precision for respiratory measurements. The authors also had experience on working with the placement of the measuring fiber in the shape of a spiral within the bed mattress [66,67].

Another interesting solution was presented in [36], where the authors achieved very good results—the accuracy in terms of heart rate measurements was 99.60±1.05%. It presented a different location for sensor placement than the one presented by the author’s colleagues in [68]. Jin Fei ([36]) focused on a real application rather than only experimental, where in [68], the authors described the implementation of a measuring optical fiber interferometer on the chest of the test subject or around the chest of the measured subject. A Michelson interferometer was used for the experimental measurements. The aim of the measurements was, in addition to monitoring the heart rhythm and respiratory rate, to measure the influence of the subject’s movement on the overall accuracy and sensitivity. The results were compared with a reference in the form of a conventional ECG sensor. The measured data obtained using the interferometric sensor differed by up to 15% compared to the ECG reference.

**Figure 2:**
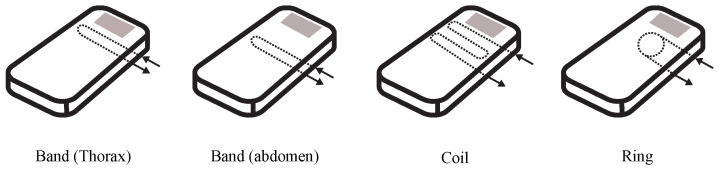
Demonstration of various configurations or implementations of an internal interferometric sensor.

Very interesting results were presented in a study [69] where the author’s team built the measuring optical fiber of the interferometric sensor directly into a mattress. The benefit was a comparison of four different configurations of measuring fibers (band (thorax), band (abdomen), coil, ring). The basic monitored parameters were respiratory and heart rate (see: Figure 2).

Another possible configuration for conducting measurements using optical fibers was presented in [70], where the optical fiber was applied along the entire length of the mattress in the shape of the letter “S” (coil). The authors’ report (based on experimental measurements) claims that they achieved an accuracy of 98.2±2% and a sensitivity of 98.4±1.1%. A similar study was carried out by the authors of reference [71], where an optical fiber was implemented in a grid-shaped mattress with a Mach–Zehnder interferometer connection.

#### 3.2.1. Fabry–Perot Interferometer Sensor

A Fabry–Perot interferometer (FPI) is generally composed of two parallel reflecting surfaces separated by a certain distance. FPI sensors can be largely classified into two categories [72,73]:

1. extrinsic,
2. intrinsic.

The extrinsic FPI sensor uses the reflections from an external cavity formed out of the fiber of interest [74]. The reflection or transmission spectrum of an FPI can be described as the wavelength-dependent intensity modulation of the input light spectrum, which is mainly caused by the optical phase difference between two reflected or transmitted beams. The maximum and the minimum peaks of the modulated spectrum mean that both beams at that particular wavelength are in-phase and out-of-phase, respectively, in the modulus of 2π. The phase difference of the FPI is simply given as shown in the Equation (1):

(1) $$\delta_{FPI}=\frac{2\pi }{\lambda }n2L,$$

where:

λ is the wavelength of the incident light,
*n* is the RI of the cavity material or cavity mode,
*L* is the physical length of the cavity.

When the perturbation is introduced to the sensor, the phase difference is influenced by the variation in the optical path length difference (OPD) of the interferometer. Applying longitudinal strain to the FPI sensor, for instance, changes the physical length of the cavity or/and the RI of the cavity material, which results in phase variations. By measuring the shift in the wavelength spectrum of an FPI, the strain applied to it can be quantitatively obtained. The free spectral range (FSR), or the spacing between adjacent interference peaks in a spectrum, is also influenced by the OPD variation. The shorter OPD gives the larger FSR. Even though a large FSR gives a wide dynamic range to a sensor, at the same time, it gives a poor resolution due to blunt peak signals [75]. Therefore, depending on applications, it is important to design the OPD of the FPI for satisfying both the dynamic range and the resolution [76].

**Figure 3:**
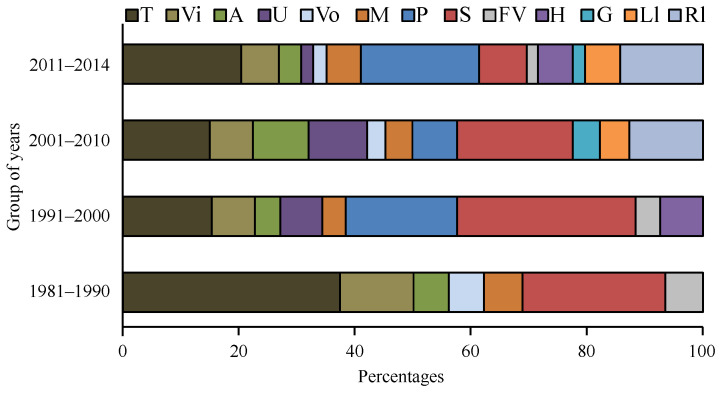
Percentage of sensing applications studied throughout some given time range.

The principles of operation of these sensors are also presented, and the development of FPI sensor applications over time is illustrated in Figure 3 [77]:

- T—Temperature,
- Vi—Vibration,
- A —Acoustic,
- U—Ultrasound,
- Vo—Voltage,
- M—Magnetic,
- P—Pressure,
- S—Strain,
- FV—Flow velocity,
- H—Humidity,
- G—Gas,
- Ll—Liquid level,
- RI—Refractive index.

#### 3.2.2. Mach–Zehnder Interferometer Sensors

Mach–Zehnder interferometers (MZIs) have been commonly used in diverse sensing applications because of their flexible configurations [78]. Early MZIs had two independent arms, which were the reference arm and the sensing arm [76]. The incident light is split into two arms by a fiber coupler and then recombined by another fiber coupler. The recombined light has an interference component according to the OPD between the two arms [79]. For sensing applications, the reference arm is kept isolated from external variation, and only the sensing arm is exposed to the variation. Then, the variation in the sensing arm induced by factors such as temperature, strain, and RI changes the OPD of the MZI, which can be easily detected by analyzing the variation in the interference signal [76].

#### 3.2.3. Michelson Interferometer Sensors

Fiber-optic sensors based on Michelson interferometers (MIs) are quite similar to the previously mentioned MZIs. The basic concept is the interference between the beams in two arms, but each beam is reflected at the end of each arm in an MI [80,81,82,83]. An MI is similar to half of an MZI in configuration. Thus, the fabrication method and the operation principle of MIs are almost the same as MZIs. The main difference is the existence of a reflector(s). Since MIs use reflection modes, they are compact and handy in practical uses and installation. Multiplexing capability with parallel connection of several sensors is another beneficial point of MIs. However, it is essential to adjust the fiber length difference between the reference arm and the sensing arm of an MI within the coherence length of the light source [76].

#### 3.2.4. Sagnac Interferometer Sensor

Sagnac interferometers (SIs) have been recently applied in numerous various sensing applications owing to the advantages of their simple structure, easy fabrication, and environmental robustness. An SI consists of an optical fiber loop, along which two beams propagate in opposite directions with different polarization states. The input light is split into two directions by a 3 dB fiber coupler, and the two counter-propagating beams are combined again at the same coupler.

Unlike other fiber optic interferometers, the OPD is determined by the polarization-dependent propagating speed of the mode guided along the loop. To maximize the polarization-dependent feature of SIs, birefringent fibers are typically utilized in sensing parts. The polarization is adjusted by a polarization controller (PC) attached at the beginning of the sensing fiber. The signal at the output port of the fiber coupler is governed by the interference between the beams polarized along the slow and fast axes. The phase of the interference is simply given by Equation (2) [84]:

(2) $$\delta_{SI}=\frac{2\pi }{\lambda }BL,B=\left|n_{f}-n_{s}\right|,$$

where:

*B* is the birefringent coefficient of the sensing fiber,
*L* is the length of the sensing fiber,
and $n_{f}$ and $n_{s}$ are the effective indices of the fast and slow modes, respectively.

In general, the sensor is based on a Sagnac interferometer. The birefringent fibers (HBFs) or polarization-maintaining fibers (PMFs) are chosen as the sensing fibers to acquire a high phase sensitivity. For temperature-sensing applications, the fiber is doped to have a large thermal expansion coefficient, which induces high birefringence variations when measuring other parameters such as strain, pressure, and twist [85]. However, the high birefringent characteristics of the HBFs and PMFs can depreciate the sensing ability due to their strong temperature dependency [76,86,87,88,89,90,91,92].

An extensive discussion of the chronology of the development of Fabry–Perrot interferometric (FPI) sensors and their use has be thoroughly discussed in a review paper by Islam et al. [77].

### 3.3. Intensity Sensors

Another type of optical fibers used for the purpose of sensing the vital functions of the human body are the so-called intensity sensors, presented and applied by the authors of reference [93], where a multi-mode optical fiber is attached under the patient’s body in order to sense the patient’s respiratory activity. The obtained results showed a very low error rate of ±1 breath per minute. It is also important to mention that the authors also presented a similar issue in [94], where they used a gradient multi-mode optical fiber in order to make the sensor, which they attached to a plastic substrate. The constructed sensor was placed under the patient’s body in the chest area. The entire sensor was designed so that it could be used to monitor the patient’s respiratory activity in the MR environment. The measurement results were based on 20 tested subjects (volunteers), where the resulting error rate of the sensor was, according to the authors, ±2 breaths per minute. The implementation of this sensor within a mattress has been further presented in publications [50,95], where the results of the study were based on 100 participants. The most favorable error rate was ±2.12 breaths per minute, and 5 volunteers had an average breath detection error rate of 5.79%.

In [96], Yu et al. explored the use of seven major fibers in conventional industries and healthcare. The new optical fiber torsion sensor was designed using a tapered seven-core fiber and was shown to measure torsion angles with an adjustable sensitivity that could reach up to 1 nm/°. In addition, the proposed torsion sensor enabled an estimation of the rotation direction with stable power. Based on the interference between the central core and the surrounding nuclei in the seven core fibers, an optical fiber interferometer was designed using a conventional sandwich structure to monitor the vital functions and activities of bedside patients. Respiratory signals and bed/body movement activities could be detected using this seven-core fiber-based interferometer.

In reference [97], Spillman Jr. and his colleagues presented the results of their research focused on the development of a “smart” bed for undisturbed monitoring of a patient’s respiration, heart rate, and movement using spatially distributed integrating multi-mode optical sensors. Their research focused on enabling greater automation of patient care, which is a particularly important issue for the elderly population, which is a fast-growing fraction of a large part of the world’s population [97]. Two spatially integrated fiber optic sensors were investigated, one based on inter-modal interference and the other based on the mode conversion. The sensing fiber was integrated into the bed, and the test subjects were monitored at various positions. The outputs from the sensor were then correlated with subject movement, respiratory rate, and heart rate. The results showed that the inter-modal sensor could detect the patient’s movement and respiratory rate, while the mode conversion sensor enabled patient movement, respiratory rate, and heart rate detection.

### 3.4. Camera-Based Systems

Vital sign monitoring using camera systems (CS) can be challenging for people with darker skin tones (darker complexion), in low-light conditions or while the individual is moving in front of the camera. In [98], Kumar and his colleagues proposed a distance PPG, which is a new algorithm for estimating vital functions based on a camera. This is a new method of combining skin color changes from different monitored areas of the face using a weighted average, where the weights depend on blood perfusion and the intensity of incident light in the area to improve the signal-to-noise ratio (SNR) from the camera estimation. The researchers also contributed a new automatic method for determination of weights based on only a subject video recording.

Yu et al. (see: [99]) developed an in-depth analysis technique to monitor the user’s respiratory rate, sleep position, and body movement during sleep without any physical contact. The researchers proposed a cross-sectional method to detect the user’s head and torso from a sequence of depth images. Eight subjects were tested. These subjects were asked to change their position on the bed (lying down and lying on their side) after 15 breathing cycles. This study is important for providing contactless technology for measuring multiple sleep conditions.

Bartula and colleagues [100] designed a camera-monitored system for reliable measurements of respiratory rate without contact with the body. A computationally efficient algorithm for extracting raw respiratory signals from a video stream was developed and implemented. Another camera offers easy access to motion information in the analyzed scenes. The performance of the sensor system was evaluated using data obtained from healthy volunteers as well as using a mechanical phantom in laboratory conditions covering a wide range of measurements.

### 3.5. Pressure Sensors

One of the most important parts of monitoring vital signs is the physical principles of various sensors, strain gauges, or accelerometers, which focus mainly on the hospital bed. Therefore, the most common are pressure sensors with a diaphragm, which can take various forms. While in previously used systems, the diaphragm diameter was on the order of tens or hundreds of millimeters and the working stroke was tenths of millimeters to millimeters, modern sensors (e.g., piezoresistive sensors) have a membrane made of a hard brittle material with a diameter of barely a few tenths of a millimeter, and the diaphragm deflection is essentially zero [101,102].

#### 3.5.1. Resistance Sensors

Resistance sensors are based on the change in resistance of a conductor during its deformation, most often when the length and cross-section change. The measuring resistors are assembled into a full or half Wheatson bridge. It deforms the meandering element, which is firmly attached to the deformed pad. The disadvantage is the dependence of the resistance on the temperature, which must be compensated [103].

#### 3.5.2. Piezoresistive Resistance Sensor

Piezoresistivity is a phenomenon that began to be used technically in the 1960s. Monocrystalline silicon is modified by the addition of trace elements (acceptors) so that its resistivity is significantly dependent on mechanical stress. This dependence is approximately 30 times more pronounced than with metal foil strain gauges. Depending on the choice of the acceptor, *P* or *N* conductivities can be achieved, which also differ, among other things, by the sign of the coefficient of resistance to mechanical stress (K-factor). The measuring member of the piezoresistive sensors is a mechanically stressed high-resistance silicon wafer on which conductive paths, usually arranged in a Wheatson bridge, are formed by the diffusion of acceptors. The silicon measuring plate is usually soldered to a glass support plate, which is then glued to a metal base made of a special alloy with the same thermal expansion (e.g., forge) [102,104,105,106,107,108].

Piezoresistive sensors are stable for a long time, providing a highly usable signal. They also have low hysteresis and very good reproductibility of measurements. The disadvantage is the high sensitivity of the silicon wafer and subtle supply conductors to any aggressive substances or moisture contained in the measured medium [109].

**Figure 4:**
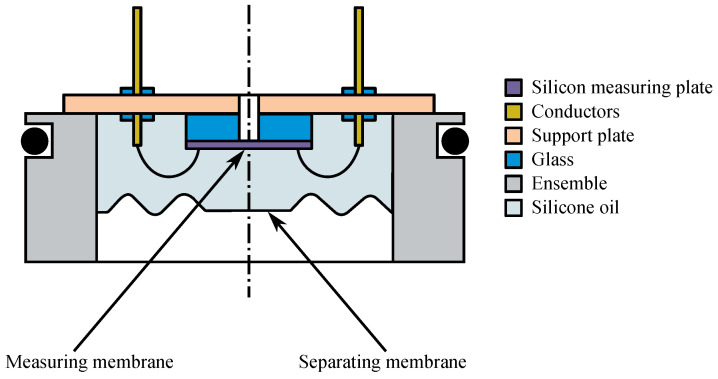
Profile of a piezoresistive sensor with a separating membrane.

In Figure 4, a profile of a piezoresistive sensor with a separating membrane [102] is presented.

#### 3.5.3. Piezoelectric Sensors

In some types of crystals, an electric charge is created by mechanical deformation. Crystals of quartz and barium titanate, for example, are used for measuring purposes, where cuts are made on surfaces that are specifically oriented to the axis of mechanical stress, and the surfaces are provided with metal electrodes [101,110]. In practice, two crystals arranged in the shape of a disk in a so-called piezoelectric twin are frequently used. The disks are connected electrically in parallel (their charges add up) and mechanically in series. They are in a pre-stressed state so that the dependence between the pressure and the change in the piezoelectric charge q on their electrodes is linear. The pressure is applied to the rigid middle part of the diaphragm, which at the same time provides the initial mechanical pre-stress. These materials enable measurement at temperatures of up to about 550 °C. At higher temperatures, the crystal loses its piezoelectric properties. However, other crystalline materials, as well as ceramic or polymeric materials, are also selected for the production of the sensors [101].

The main advantages of piezoelectric pressure sensors are their small size and lightweight properties, which lead to good dynamic properties. Miniature sensors as small as a few millimeters and with a time constant on the order of microseconds are designed to measure dynamic pressure profiles; e.g., pulsations in internal combustion engines and compressors [101,111]. One of their disadvantages is their temperature dependence and more difficult signal processing, as special cables and impedance-matched amplifiers with high-resistance inputs are required. Another disadvantage is their inability to measure in a static state because the charge created by introducing a deformation into the crystal quickly discharges and disappears [101,112].

By incorporating an impedance transducer into the housing of the piezoelectric pressure sensor, where the disadvantage of the need for using special cables with a high insulation resistance (expensive) is eliminated, cable noise is reduced, but at the same time, the upper operating temperature limits are additionally reduced [110].

### 3.6. Strain Gauges

Strain gauges are used for universal and accurate measurements of deformations, forces, pressures, and moments acting on solids [113,114]. Among the classic strain gauges, it is possible to differentiate between metal resistance strain gauges [113,114,115].

The first strain gauge (English strain gauge), hereinafter called a deformation transducer to change resistance, was first practically built in 1938 and has been used in many areas of the industry ever since. For example, they are used by builders, architects, designers, the automotive and engineering industries, and for pressure and force measurements in pneumatic equipment [114,116,117]. At first glance, they are invisible, and they appear in heating and ventilation, where miniature integrated silicon strain gauges are hidden in the sensors to measure pressure and force. They are found everywhere where it is necessary to directly electrically measure or monitor the deformation of a solid-state object. A separate type are strain gauge sensors (strain gauge cells), which can be found in most common weighing devices.

The first strain gauges were metal wires [116]. In 1952, strain gauges were created using foil, which are still used in large quantities today when they compete with semiconductor (silicon) strain gauges. Although silicon strain gauges are characterized by up to 60 times greater sensitivity, i.e., a change in resistance to a change in length, on the contrary, linearity and accuracy are trumped by the foil gauges. This means that foil strain gauges are used wherever it is necessary to measure with increased accuracy [102,115,116,118].

In practice, strain gauges are used not only in industry (measuring deformations of objects, etc.) but also in medicine—for example, for monitoring vital functions using a bed sensor [113,114,115].

**Figure 5:**
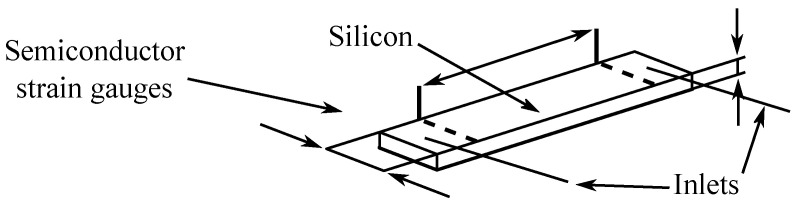
Semiconductor strain gauge.

More precisely, electric semiconductor strain gauges are based on the piezoresistive phenomenon of piezoresistance, i.e., on the change in electrical resistance depending on the deformation of a semiconductor crystal (e.g., silicon single crystal, germanium) [119,120], as can be seen in Figure 5.

Semiconductors can change the conductivity across a very wide range (by 6 to 8 orders of magnitude) due to external physical phenomena (pressure, tension, temperature, light) or by adding a small number of impurities (foreign atoms) to the pure substance of the semiconductor [102,121].

Semiconductor strain gauges consist of a strip cut from a single crystal of a semiconductor (silicon, germanium, etc.) contaminated by the diffusion of another material [121,122]. The mechanical stress of the crystal lattice significantly affects the mobility of the charge carriers and thus the specific resistance of the strain gauge material. Depending on the type of contamination, the resistance of the deformed material increases or decreases [120,121,123].

### 3.7. Micro Electro-Mechanical System (MEMS) Accelerometers

Accelerometers are used to measure static gravitational acceleration, which allows for the determination of the deflection angle of the measured object from the gravitational acceleration vector. They are also used to measure the dynamic acceleration due to shocks, motion, or vibrations with low amplitudes and low frequencies, which reaches several tens of Hz.

The accelerometers can be applied, for example, in medical and sports technologies and in cameras, smartphones, remote controls, and navigation systems. The basic principle is based on the conversion of the acceleration (m·s^−2^) to the force *F*(N), utilizing the mass *m* (g), which acts on the sensing element according to Equation (3):

(3) $$a=\frac{m}{F}.$$

Acceleration is a vector quantity; therefore, we measure the magnitude and direction of action with respect to the size and meaning of the vector components in the individual axes [124,125].

If no external acceleration is applied to the accelerometer, the device will only measure the gravitational acceleration. The triaxial accelerometer measures the magnitude of the acceleration in three axes perpendicular to each other. Based on the magnitude of these values, it is possible to determine the position of the sensor concerning the action of the gravitational acceleration vector *g* [124,125].

The basic parts of accelerometric sensors are as follows [124,125]:

- seismic matter;
- sensor element;
- coverage.

Sensing can be performed by changing the resistance of strain gauges, by changing the capacitance through the membrane or the position of the mass, by changing the inductance by moving the core, or by deforming the piezoelectric material [124,125,126].

The basic types of accelerometers are as follows [124,125,126]:

- MEMS capacitive accelerometers,
- MEMS piezoelectric accelerometers,
- MEMS piezoresistive accelerometers.

Capacitive accelerometers that use MEMS technology are the most inexpensive, the smallest, and the most common sensors of this type. The principle of their function consists of placing the material on flexible hinges. Due to the force acting on the sensor, the mass moves on the hinges, which causes a change in the distance between the plates of the differential capacitor, thus affecting the change in capacity [124].

Capacitive MEMS accelerometers are commonly used in wearable electronics, mobile devices, and in general consumer-grade electronics [127]. The shortcomings of MEMS circuits include their lower measurement accuracy, especially in the case of measuring higher amplitudes and frequencies [124,125,128].

In this paper [129], the authors describe the fundamentals of the operation of capacitive principle MEMS accelerometers manufactured using CMOS technology. It also covers the features and disadvantages or problems involved in making these MEMS multi-layer semiconductor structures; for example, deformation of the base substrate or noise. The authors performed a series of successful experiments on this type of accelerometer.

The authors of reference [127] deal in detail with the possibilities of optimizing the individual parameters of the MESM accelerometer, including a detailed description of the influence of individual components. In this paper, they present the difference and relationship between the design optimization of a capacitive MEMS combination accelerometer device with a composite beam and the sensitivity of the device, such as the beam width, beam length, and mass width. Based on the analysis, an optimized design of the MEMS combination capacitive accelerometer device is proposed.

### 3.8. Radio Frequency Identification

Radio Frequency Identification (RFID) is primarily intended for the transmission and storage of data via electromagnetic transmission from a transmitter to any radio frequency (RF)-compatible circuit. The data are sent and read from RFID tags using a sensor using radio waves. The RFID system consists of a reader that can communicate with RFID tags, which transmit data that the RFID reader can read using a predefined RF and a protocol known to both the reader and the tags a priori. The data collected from the tags are usually sent from the sensor via a communication interface (wired LAN or wireless WLAN) to the host computer system [124,125].

In passive systems, which are probably the most common, for activation, the RFID sensor must generate an electromagnetic field (EM), the right amount of energy to “wake up” the tag, and provide energy for the response via a backscatter. The reader and tag are magnetically paired by both resonating at the same frequency for maximum transmission power. Resonant circuits consist of an inductor and a capacitor. In other words, the reader generates an EM field, which supplies the chip inside the RFID tag. If the transponder is in the electromagnetic field of the RFID reader, then data exchange takes place; i.e., new data can be loaded and saved [125].

Active systems have batteries that are placed in the tag. The advantage is to increase the effective range of the tag and support other functions that passive tags do not have, such as sensing physical quantities such as temperature, pressure, humidity, etc. These tags are collectively referred to as transponders and dataloggers. Active systems can transmit data over longer distances (up to 100 m), transmit a strong signal, and reach a large reading range. The disadvantage is the increase in the size of the tag and the need for frequent battery replacement [125].

There are also intermediate stages of so-called semi-active and semi-passive systems, which have their source of energy but do not function as a separate transmitter. The RFID transponder is supplied with electricity from the battery, where it is not necessary to take away the power of the electromagnetic field. However, the response is given in the form of field modulation, which is not repeatedly amplified.

The RFID structure is as follows [92,125,128,130]:

- RFID tag: The inside of an RFID transponder consists of an integrated circuit (IC) connected to an antenna, which is either printed, folded, or etched—typically a small wire coil that transmits data to an RFID sensor (sometimes called an interrogator). Tags are also sometimes called “transponders” or “inlays”, although technically, an inlay is a tag attached to a backing strip. The chip and antenna are very sensitive to mechanical, thermal, and chemical influences. For this reason, they are protected by at least a simple protective foil or a layer of paper, or the electronics are wrapped in a laminate card or a plastic cover.
- RFID sensor: An RFID sensor (formerly called an RFID interrogator) is a radio frequency (RF) transmitter and receiver controlled by a microprocessor or digital signal processor. The sensor converts electromagnetic waves into a more usable form of data. The information obtained from the tags is then sent by the sensor via the communication interface to the host system, where the data can be stored in a database and later analyzed. The sensor, using a connected antenna, generally an RFID reader, reads the data from the tags and then passes it to a computer for processing.
- RFID antenna: The data are stored in the memory of the integrated circuit and transmitted to the sensor through the antenna.

In Table 3 and Table 4, the reading range and the frequency domain of the RFID tag are presented.

Usability of RFID:

- Real-time monitoring of respiratory rate [131,132,133,134];
- Detection of sleep apnea [131,132,133,134];
- Heart rate monitoring [130,134];
- Body position detection (pressure ulcer monitoring) [133,135];
- Bed-wetting alarms [136];
- Monitoring the presence/absence in bed [137,138];
- Restless leg syndrome (RLS) [132];
- Falls [137,138];
- Longer periods of inactivity (caused, for example, by fainting, unconsciousness, or even death) [137,138].

In reference [134], Zhao et al. introduced an RFID-based CRH system for contactless monitoring of heart rate and respiratory rate. This technique uses an empirical decomposition procedure to estimate a subject’s heart rate and respiratory rate.

Wang et al. [139] introduced a technique called RF-ECG that also uses passive RFID technology to monitor heart rate variability. The RF-ECG uses passive RFID tags attached to the subject’s chest and requires the object to be present in front of the antenna in a static position. This technique uses the phase values of the signals reflected from these tags, which transmit information about the heart rhythm.

Yang et al. [140] introduced a technique called Autotag for the detection of respiration and apnea using phase values from the RFID tags attached to a subject’s chest area. This technique uses a repetitive variation model of the autoencoder to detect apnea.

Acharya et al. [141] proposed a technique based on passive RFID tags for the detection of respiratory activity (respiratory and non-respiratory events). This technique uses two RFID tags, one as a reference tag placed on the arm to capture signal artifacts and the other placed on the abdomen to capture breath movement.

Hou et al. [142] introduced a technique called TageBreathe, which also uses RFID tags for breath monitoring. The TagBreathe uses phase values obtained from multiple tags attached to the subject’s chest area and uses frequency domain analysis of the captured signal to capture the signal. They designed a non-invasive breath monitoring system to monitor breath with a high accuracy, even in the presence of multiple users. In this system, RFID tags are built into yarns that can be used to make RFID garments. When a person is wearing such clothes, he or she will not be disturbed by these light, thin tags. However, the labels are still attached to the body.

For breath monitoring, the received signal strength indicator (RSSI) values are subtracted from each tag, and for body movement monitoring, the CNN algorithm is used to detect which part of the body is causing the backscatter of the signal reflection backscatter. The backscattered signals from each tag are divided into two types of body movements [132]:

1. Gentle chest movements caused by breathing;
2. Strong body movements such as twitching of the limbs.

The markers are placed on the bed linen to obtain backscattered signal changes caused by the two above-mentioned main movement types [132].

### 3.9. Non-Contact RF Vital Sign Sensor

In recent years, modern methods based on the principle of propagation and reflection of electromagnetic waves have begun to be used for the non-invasive measurement of basic vital functions. The use of radar can be included among the most well-known principles. Radar is a device used to detect objects within their range and to measure the distance and speed of these objects. The word “radar” itself was created by combining the English words “radio detection” and “ranging”.

The development of single-chip radars in recent years has made it possible to use these radars in passenger cars in reversing assistants to control blind zones behind the vehicle, to control the distance from other vehicles, and to check for the presence of children in the car seat. Another possibility of using micro-power single-chip radars is offered in medicine. Research and pilot projects on the possibility of implementing radars for contactless monitoring of breath and heart activity are being carried out [143,144,145].

Bibb et al. [144] developed technology for measuring vital signs and measuring lung water using a wireless microwave antenna monitoring system. The proposed system uses a unique sensor/antenna to connect the electromagnetic energy and is also equipped with a mobile application to display the processed values in real-time and to facilitate use by mobile users. New RF components were used, eliminating the need for expensive and bulky network analyzers. The developed system is more suitable for clinical use and remote monitoring of remote patients. The accuracy of the developed wireless system was verified by comparing the results with traditional network analyzer measurements, and excellent agreement was observed.

The principle of radar operation is that the radar sends an electromagnetic wave through a transmitter. The transmitted wave propagates through space and bounces off the objects it hits. The reflected wave, sometimes called an echo, is then received by the receiver. The received signal is then further evaluated in order to obtain information about the object from which the wave bounced. In this way, it is possible to obtain information about the position, distance, and relative speed of the object concerning the radar that detected the object.

The principle of operation results in four basic radar blocks [146]:

1. Transmitter—The task of the transmitter is the appropriate form of an electromagnetic wave and its subsequent transmission with sufficient power.
2. Antenna—The range, sensitivity, and directional resolution of the radar depend on the quality of the antenna. High-directional antennas are usually used for radars. The direction and height of the detected target are determined from the antenna settings. Often, a single antenna is used for both transmission and reception.
3. Receiver—The task of the receiver is to capture the reflected signal and amplify it to a sufficient level so that it can be further processed. The strength of the captured signal depends mainly on the distance to the target. When capturing echo from a near target, the signal may be relatively strong, while signals reflected from more distant targets will be very weak. The receiver must therefore be able to process signals with a large dynamic range. Due to the processing of very weak signals, little noise is required from the receiver circuits.
4. Signal processing block—In the signal processing block, the evaluation of the received signal is performed. Calculation of the distance from the detected target is used to evaluate its speed and possibly suppressed unwanted reflections (clutter), for example, from the lateral lobes of the antenna or the surrounding terrain.
5. Duplexer—If the radar uses one antenna for transmission and reception, a duplexer is a necessary part. The purpose of the duplexer is to prevent the transmitted signal from penetrating the input of the receiver and the received signal from penetrating the transmitter.

**Figure 6:**
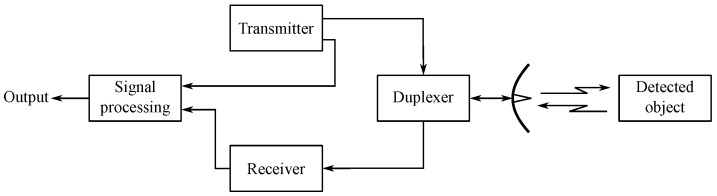
Basic block diagram of a radar with a common antenna for transmitting reception.

A block diagram of a radar with a common antenna for transmission and reception is shown in Figure 6 [146].

Radars usually operate in the 2.4 GHz, 2.76 GHz, or 10 GHz frequency bands. Exceptionally, higher bands of the order of 60 GHz are also used [147]. A radar can be any system that sends out radio waves that reflect off surfaces and return to be detected by the same device. “Radar” was originally short for “radio detecting and ranging”. The purpose is to figure out the presence, speed, distance, or direction of objects, especially vehicles. For example, radar can be used to track airplanes. This is similar to the way that submarines track other underwater objects, except that underwater sonar uses sound instead of radio waves.

From a design point of view, it is possible to differentiate between mono-static radars and bi-static radars. Mono-static radars have a transmitter and a receiver in one place. Mono-static radar transmitters and receivers often use a common antenna that is alternately switched for transmission and reception. The bi-static radar receiver and transmitter are separated from each other and can even be located in different places. Another division of radars is given by the form of the signal with which the radars work. According to this criterion, we distinguish between pulse and continuous radars. Continuous radars can be further divided into Doppler radars and modulated signal radars [145,147].

Doppler radar belongs to the group of continuous radars. Continuous radars are called CWs (continuous wave radars). The Doppler radar transmitter transmits a continuous harmonic signal with a constant frequency. This signal is reflected from surrounding objects and is then picked up by the receiver. However, if any of the objects causing the signal reflection move at a certain non-zero speed relative to the radar, a Doppler shift occurs. The magnitude of this shift is determined for the received signal, and the speed of the given object is subsequently determined from it. Based on the principles of Doppler radar operation, it is clear that this technology allows for the detection of only moving targets. However, it is not possible to determine the distance of the detected objects. The reason for this is that it is not possible to measure the time between sending a signal and then receiving an echo. In order to determine the distance of the detected object, continuous radars are supplemented by frequency or phase modulation [145,147].

Another type is a frequency-modulated continuous radar, where in order to obtain information about the distance of the monitored object, it is necessary to adjust the course of the transmitted signal so that it is possible to determine the delay between the transmission of the signal and the subsequent capture of the echo. One of the possibilities of adjusting the transmitted signal is frequency modulation. Radars using a frequency-modulated continuous signal are referred to as frequency-modulated continuous wave (FMCW) radars. The transmitted signal is usually modulated by a saw-tooth waveform, which allows for easy determination of the detected delay. This technology is often used in radar altimeters [145,147].

Pulse radars differ from continuous radars because they do not transmit and receive continuously but instead emit a very short electromagnetic pulse of a relatively high power. After the pulse is sent, the receiver is activated, trying to pick up the reflected signal. After a certain time interval, the receiver is switched off, and another pulse is sent. From the time interval between the sent and the received signal, it is again possible to determine the distance of the monitored object. Radars using pulse compression also fall into this category [145,147].

Doppler radars are used to measure speed. Pulse radars or CW radars are used to measure distances. By combining signals from these radars, it is possible to accurately monitor the movements of the tissue interface during cardiac activity as well as the movement of the lungs, which represents the patient’s breathing [145,147].

The basic principle of measuring distance using radar is to determine the time it takes for the transmitted electromagnetic wave to travel the distance from the antenna to the target and back. Based on this time and the known speed of propagation of an electromagnetic wave through space, we can easily calculate the distance of the target from the antenna according to the relation expressed in Equation (4) [146]:

(4) $$R=\frac{c}{2}\cdot \Delta t$$

where:

*R*—distance between the antenna and the target,
*c*—speed of the electromagnetic wave propagation,
Δt—time between the sending wave and the echo capturing.

For example, in reference [148], Chang and Chen represent vital sign processing circuits for simultaneous elimination of DC and suppression of out-of-band interference without any digital signal processing or with the aid of an ultra-wide band (UWB) pulse radar system algorithm. The self-balanced MOS diode (SBMD) has been designed as a stable and balanced pseudo-resistor applied under the servo feedback loop in the vital radar receiver to act as a high-pass filter (HPF) with an ultralow corner frequency below 0.5 Hz to remove unwanted reflected signal interference. An input DC offset voltage from the innate offsets of the circuit. A Chebyshev low-pass filter (SCF) with a third-frog topology used as the subsequent phase was taken to suppress external sounds, creating an integrated vital sign-processing circuit with frequency bandwidth and its integration into the radar module to verify its viability.

## 4. Sleep Quality Monitoring

Aubert and Brauers applied and described in their work a solution [149] where a single non-contact mechanical sensor is applied for estimation of the three vital signs during sleep, including heart rate, respiratory rate, and activity index associated with body movements. The robust estimates were carried out in 30-s epochs. Data processing was performed using standard DSP dynamic packet state (DPS) techniques, which led to an integrated solution for working with body movement artifacts. The algorithms have been described and evaluated on hundreds of nocturnal corpora collected from real recordings obtained from both healthy subjects and patients from the sleep laboratory.

Another interesting finding was presented by Clemente et al. in [150], where they introduced a new real-time contactless sensor system called Helena, which can be mounted on the bed frame to continuously monitor sleep activities (bed entry/exit, movement, and position changes), vital signs (heart rate and respiratory rate), and falls from bed in real-time time and in a ubiquitous way. The intelligent sensors mounted to the bed enabled the researchers to observe bed vibrations generated by body movements and characterizes sleep activities and vital functions based on advanced signal processing and machine learning methods.

In reference [151], Jia et al. used geophones in order to sense bed vibrations caused by ballistic force. This method has been proven to have great potential for monitoring heart rate during sleep. It does not require a special mattress or sheets. The user is able to move and change position freely during sleep. The authors proposed a new system called VitalMon for respiratory and heart-rate monitoring purposes, even in the case of sharing a bed with another person. In such cases, the vibrations of both co-sleepers are mixed, but the VitalMon separates the two heart rate signals first and then distinguishes the respiratory signal from the heart rate signal for each person. This algorithm separates the heart rhythm and relies on the spatial difference between the two signal sources concerning each vibration sensor.

In [152], Prakash and his colleagues presented the implementation of a set of sensors and devices for inconspicuous measurement of physiological and behavioral parameters indicating sleep quality; in particular, force transducers, electromechanical film, and thermocouples were used to measure the respiratory rate, pulse rate, and physical activity of a bedridden subject. A set of sensors was developed to monitor the quality of sleep in children with severe developmental disabilities. The presented technologies have great potential to provide an objective assessment of sleep quality for children in their home environment.

An interesting solution developed for sleep quality monitoring purposes has already been described above and was developed by Yu et al. and presented in reference [99]. The proposed system enables sleep monitoring based on camera sensors without any physical contact between the user and the sensor.

Unlike conventional monitoring devices, RFID scanning technology is gaining great interest due to the weight, low cost, and tag size, which are suitable for undisturbed sleep monitoring. RFID automatically identifies and tracks tags embedded in objects (such as clothing) based on electromagnetic fields. Low-level information, including received signal strength (RSS), phase value, and Doppler shift, is reported by RFID readers, which can be used for a variety of applications. For example, the collected RSSI and the phase value, which indicates the distance between the tags and the reader’s antenna, can detect when a person inhales/exhales or changes his or her position [132].

**Figure 7:**
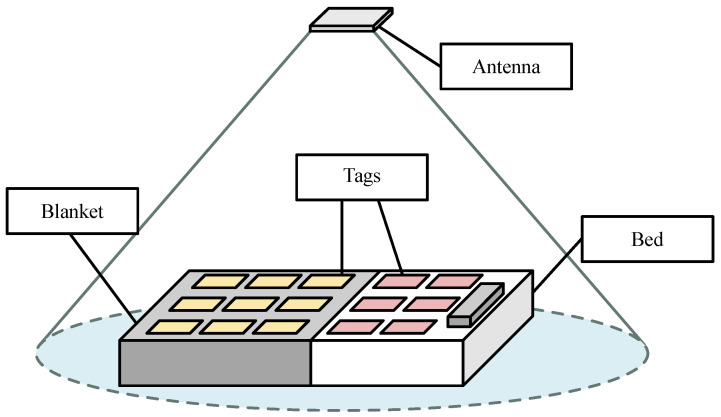
Illustration of an environment for sleep monitoring [132].

Figure 7 shows a sleep monitoring environment. Labels or tags are attached under the sheets and in blankets in a regular distribution, with a few inches on either side. The antenna is located on the ceiling directly above the bed. The distance between the antenna and the bed is 2 m, and the person lies on the bed with a breath of 60 s.

Hu et al. [132] designed a sleep monitoring system based on passive RFID technology. Their work applied RSSI values reflected from a passive RFID tag built into the bedding to capture information about breathing and body movements. A time–domain analysis was used to calculate respiratory rate, and a convolutional neural network was used to detect body movements.

Hussain et al. provided and described in their work [133] an easy-to-use, non-invasive, and inexpensive solution for overnight sleep and breath monitoring. They attached two passive tags to the person’s T-shirt and use the RFID reader and one antenna to collect RSSI values, which means that the measurement will be effective only if the person is in one position. This problem can be solved by adding several more antennas.

In [135], the authors reported on wireless identification and sensing platform (WISP) tags integrated into a mattress, where they are not in direct contact with the body in order to detect movement during sleep. Reading from WISP tags, however, is not sensitive to light movements such as breathing.

In [153], Sharma and Kan used a passive RFID tag for sleep scoring. In their work, a single RFID tag was attached to a subject’s chest area,. The authors also applied a support vector machine (SVM) to recognize various sleep activities such as throwing, body jerking, and rotation.

A very interesting system for remote sleep quality monitoring is an ambient intelligence system. The platform is fully based on passive radio frequency RFID distributed around the bed and integrated into the user’s clothing. Sleep status identification (quiet sleep, out of bed, prolonged absence, and falls) and sleep position classification (on the abdomen, back, left/right) are obtained with combined digital and analog processing of backscattered signals from markers with an accuracy close to 100%, as evaluated in laboratory and real conditions-based experiments [137].

**Figure 8:**
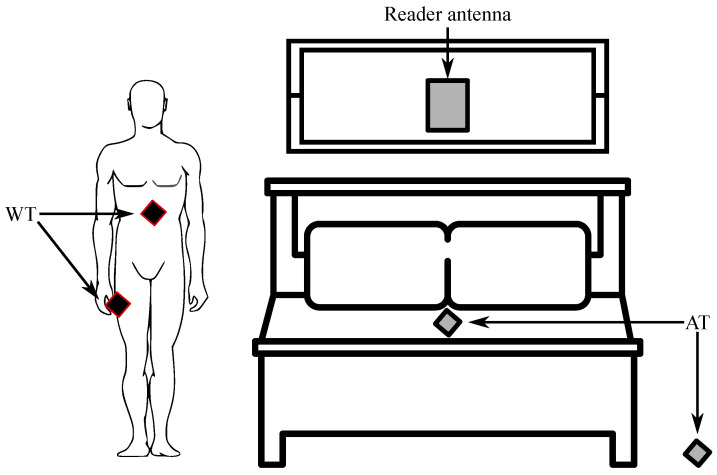
NIGHT-Care platform [138].

An interesting project is NIGHT-Care: a passive RFID system for remote monitoring and control of an overnight living environment. The platform is presented in Figure 8 and uses miniaturized wearable tags (WTs) integrated into the garment, conventional environmental tags (AT) scattered in the environment, a long-range UHF RFID reader, a physical processing software engine for real-time processing, and a web graphics processor with warning modules. When processing electromagnetic signals generated by the interaction between the subject and the environment, the system detects and reports the presence (absence) of the user in bed, his jerky movements and movement patterns, accidental falls, prolonged absences from the bed, and longer periods of inactivity (caused by fainting, unconsciousness, or even death) [138].

The tag’s response to the reader’s request is subject to ambient modulation, in the sense that the strength of the backscattered field is adjusted by the position of the human body between the tags themselves. In addition, in the case of specific body positions relative to the environment, the child tag may be completely obscured so that it will not be able to respond to the reader’s request. For example, if the object is lying on the bed, the tag under the mattress is completely shielded and will not react, while others on the floor can communicate freely. On the contrary, it will happen if the patient falls to the floor. Patient activity during the night can therefore be recognized by processing the signals received from the tags. In more detail, the IDs of the responding tags can be used to identify the patient’s condition (whether in bed, falling, or outside), while the processing of electromagnetic field strength (RSSI levels) is reflected by the responding tags. Therefore, the signals detected by the reader can be used to extract movement and specific positions during sleep [137].

**Figure 9:**
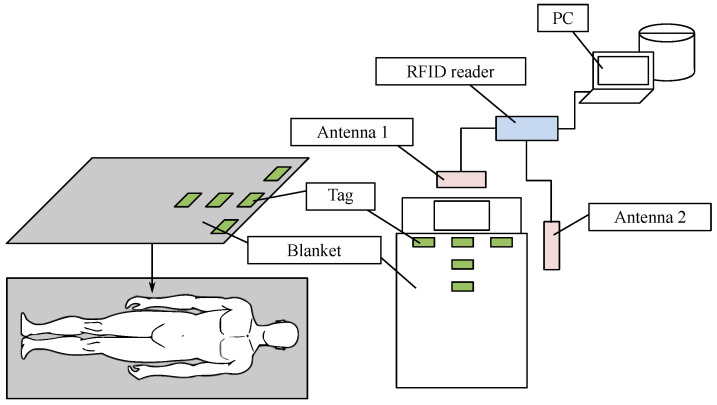
Overview of the proposed system [131].

Another remarkable solution is a non-invasive and quick respiratory-rate monitoring system at bedtime using passive RFIDs. The system is illustrated in Figure 9 and estimates RR (respiratory rate) by placing antennas close to the user and attaching RFID tags to the blanket instead of connecting the device directly to the user. This system has three main characteristics: tt monitors the respiratory rate in real-time, it records changes in the respiratory rate for a long time, and the patient does not have to wear any equipment. Bed sleep RR was estimated using RSSI and phase values obtained from RFID tags. The results of the evaluation confirmed that the system can estimate the respiratory rate regardless of the user’s posture, body type, gender, or change in respiratory rate [131].

## 5. Vital Sign Monitoring Challenges

Monitoring vital signs play a key role in both the prevention and diagnosis of various health problems. As more and more people pay attention to their health, various measuring systems are being developed. The problem with measuring systems is that they can be uncomfortable for patients because they require physical contact with the body. Those that must be contactless, on the other hand, are prone to various interferences and artifacts and are therefore often less effective [154].

There are many algorithms in the literature for measuring human vital functions [155,156]. Vital signs, as well as biomedical data, are prone to various internal and external artifacts that could be related to some interferences. In addition, measuring vital signs while driving is a different challenge because there are different body movements. The occurrence of interference in the examined or recorded data has a very negative effect on their quality and complicates the whole analysis process [157].

One of the important interferences is that which is connected to the power line; that is, 60 Hz for the USA and 50 for the rest of the world, including Europe. It can be easily reduced by implementing a basic notch filter.

It is possible to differentiate between inter-modal and intramodal interferences in, e.g., cECG recordings that do not affect the quality of the recording and do not interfere with the monitoring of vital signs [154].

Other interferences are strongly associated with patient’s movements or antenna design (for wireless data transmission) [154]. Also, other signals, such as those coming from the background, can cause unnecessary interference.

Table 5 summarizes the occurrence of different types of interferences in the measured signals with the use the of methods discussed in this paper. Various innovative methods are used to suppress them [157].

Biomedical signals or vital signs are very challenging to analyze due to their nature and non-stationarity (in most cases). They are also prone to the occurrence of various artifacts [154,157,158].

Signals from inconspicuous sensors are more prone to noise and artifact occurrence (as mentioned above) and are more sensitive to motion artifacts. A fusion of multi-modal sensors has been proposed by some sources in order to increase the accuracy and robustness of the measured parameters, such as:

- cECG and rPPG [42]; cECG and BCG [159].

The above are combined for the purpose of more reliable heart rate detection. Similarly, ECG and BCG have been integrated into a bathroom weight scale [160], as well as ECG, electromyogram (EMG), impedance plethysmogram (IPG), and BCG [161].

The above-mentioned multi-modal sensor fusion also allows for the extraction of other parameters that cannot be derived from a single measurement modality [2].

One negative aspect in measuring the vital functions of the human body is too high sensitivities of sensors (reduced SNR), the location of the reference arm, couplers, or larger dimensions. Mach–Zehnder, Michelson, and Fabry–Perot are among the most frequently used types of interferometers for biomedical applications [162].

Physical principles of various sensors, strain gauges, or accelerometers, which focus mainly on hospital beds, play a very important role in vital sign monitoring [163].

## 6. Discussion

One of the main reasons for writing this review paper was the necessity for continuous monitoring of key vital parameters in various settings, including hospitals as these can provide valuable information regarding the patient’s condition and thus enable early intervention in case of emergencies. Continuous monitoring of vital parameters is crucial for patient care, especially in intensive care units (ICUs), where timely detection of physiological changes can be life-saving.

The key vital parameters that are essential for monitoring include heart rate, blood pressure, arterial oxygen saturation, and respiratory rate. These parameters are critical indicators of a patient’s health status and are routinely monitored in clinical practice. Currently, devices such as electrocardiograms (ECG), photoplethysmograms (PPG), and arterial blood pressure sensors (invasive) or blood pressure cuffs (non-invasive) are used to measure these vital signs. While these devices provide reliable data, they can often be uncomfortable for patients and may restrict their movement, leading to potential discomfort and anxiety.

In order to address these limitations, innovative solutions such as sensor systems mounted just below the mattress have been developed, which offer continuous monitoring of vital signs without interfering with the patient’s comfort or mobility, providing a non-invasive and patient-friendly approach to health monitoring. This method not only enhances patient comfort but also ensures that healthcare providers receive continuous and accurate data, which are critical for making informed clinical decisions.

The advancement of contactless monitoring technologies represents the future of medicine, offering a more comfortable and efficient way to monitor vital functions. These technologies hold the promise of improving patient outcomes by allowing for early detection of potential health issues, thereby enabling prompt medical interventions. The development and implementation of such innovative monitoring systems are pivotal in advancing healthcare and enhancing the quality of patient care in hospital settings [154,157].

Although this article provides an analysis of sensors that are suitable for implementation at patient bedsides, several areas could be the subject of more extensive research. First, there is a need to further research and develop new types of sensors that can offer a higher accuracy, reliability, and longer life without significantly increasing their cost. Investigating the use of materials with better properties or more advanced technologies could lead to significant improvements in these sensors. Another potential area of research is the integration of these sensors with advanced data analysis tools and machine learning algorithms. The development of sophisticated models for the analysis of data obtained from simple and inexpensive sensors could enable more accurate and timely detection of changes in the health status of patients, which could lead to better personalization of care. It would be useful to conduct larger clinical studies aimed at verifying the effectiveness and benefits of these sensors in a real clinical setting. Research focused on the long-term follow-up of patients and their interactions with these technologies could provide valuable information about the practical benefits and possible limitations of these systems. Finally, considering the rapid development in the field of wearable technologies, it would be interesting to explore the possibility of combining bed sensors with wearable devices to supplement vital functions that cannot be obtained through bed sensors. This combination could provide more comprehensive monitoring of the patient’s health status and contribute to an overall improvement in care.

## 7. Conclusions

Vital signs are a crucial component of monitoring a patient’s condition during hospitalization because they enable the immediate detection of delayed recovery or adverse side effects. Continuous monitoring of these ensures that healthcare professionals can promptly respond to any changes in the patient’s condition.

An initial search of the literature revealed a significant gap in comprehensive research on various aspects of vital signs monitoring, particularly of the non-invasive and contactless methods. This gap highlights the need for a thorough review focused on specific aspects of measuring vital signs. Consequently, we undertook this review to address these gaps and provide a detailed overview of monitoring functions centered around the hospital bed. It focuses primarily on the monitoring of signals such as capacitive electrocardiography (cECG), ballistocardiography (BCG), and electroencephalography (EEG). Additionally, we examined the use of optical fibers and camera systems for the purpose of vital sign monitoring and the potential interferences they may be encountered during the measurements. These technologies offer a range of advantages and challenges, which we have explored in detail to provide a clear understanding of their applications and limitations. Furthermore, we explained the underlying physical principles that govern these monitoring techniques, offering insights into how they function and their potential for integration into clinical practice.

Although there is existing research addressing specific aspects of vital sign monitoring, our review consolidates this information to provide a comprehensive resource that addresses a significant topic and issue in the field. By doing so, we aim to facilitate the development and implementation of innovative monitoring solutions that enhance patient care and improve clinical outcomes.

## Figures and Tables

**Table 1:** Overview of links according to the monitoring of individual vital functions.

Vital Function	Link
Heart rate (HR)	Section 3.1 and Section 3.8
Arterial oxygen saturation	Section 3.4
Respiratory rate	Section 3.1, Section 3.8 and Section 3.4
Magnetic induction monitoring (MI)	Section 3.9
Ballistocardiography (BCG)	Section 2
Photoplethysmographic imaging	Section 3.4

**Table 2:** Comparison of the most frequently used FBG sensors.

Ref.	Meas. Var.	Sensor Properties	Sensor Placement	Sensor Efficiency
1	RR	Field of 12 FBGs	Under the mattress	More than 95% in terms of recognition rate and signal-to-noise ratio (depends on sleeping position)
2	RR	3 sensor arrays, each with 6 FBGs	On the bed under a thin sheet	Mean detection error is below 1%
3	RR, HR	Array of 12 FBGs arranged in a 3×4 matrix	On the bed under the mattress	The system showed a maximum error of ±1 breaths per minute compared to manual counting
4	RR, HR	Plexiglass sheet (95×220 mm), FBG attached with epoxy glue	Under the patient’s body	Statistical analyses of the results proved that the sensor demonstrated good measuring properties and met the basic requirements for patient monitoring
5	RR, HR	Field of 8 FBGs, sensor array was packaged onto a polycarbonate sheet	Placed on top of the mattress	Mean error of less than 1 bpm, more than 95% accuracy rate for pulse and respiratory rate monitoring for patients lying in beds
6	RR, HR, presence or absence of the patient in the bed	Field of 12 FBGs	Placed either beneath or on the mattress	For respiration rate, the system showed a maximum error of ±2 breaths per minute compared with manual counting, heart rate detected by the FBG optical bed sensory system, which is comparable to that measured by an off-the-shelf pulse oximeter
7	HR	Field of 6 FBGs	Placed on top of the mattress, under the patient’s body	The best heart rate sensor array was placed under the chest and abdomen, where the average and standard deviation of the error were 0.049 and 0.019, respectively
8	HR	3 arrays of 6 FBG sensors	FBG sensors embedded in a mattress	The mean error of estimation obtained was below 1 BPM

**Table 3:** Reading range of RFID tag.

Connection Type	Frequency Range	Distance	Description
Close coupling	1 Hz–30 MHz	0 to 1 cm	The position of the transponder relative to the reader must be precisely defined during reading. The amount of energy transferred increases in proportion to the frequency. The transmission can be safely realized by an inductive or capacitive connection.
Remote coupling	100 kHz–27.125 MHz	Up to 1 m	Data transfer is realized by an inductive connection. The transmitted energy from the magnetic field of the reader is sufficient for the operation of the chip (transponder supplied passively).
Long range	Microwave spectrum	1 to 10 m	Data transfer is realized through a “backscatter”; it is sufficient only to send a wake-up or sleep signal. Support batteries are required to supply the transponder chip with RFID power and to store the stored data. Typical use in toll systems.

**Table 4:** Frequency domains.

Frequency Band	Frequency	Note
Low frequency (LF)	125 kHz	Low data rates and distances;
		Cost-effective and easy to use, without application and fees;
		Used for near field electromagnetic waves;
		Passively supplied with energy from an inductive connection;
		Relatively resistant to metals or liquids;
		Used in animal identification.
High frequency (HF)	13.56 MHz	High data transfer rate and high clock speed;
		Only a few antenna windings are enough to build, etched or printed antennas are also possible.
Ultra-high frequency (UHF)	860–950 MHz (divided into sub-bands)	They can be detected at close or far distances from the reader and can therefore be used for a wide range of applications;
		Highest data transfer rate;
		Production is more cost-effective.

**Table 5:** Interferences occurring in the measured data during vital sign monitoring.

	cECG	BCG	CS	FBG	EEG
Power line	High	Low	Low	Low	High
Motion	Medium	Medium	High	Medium	High
Background noise	Low	Low	Low	Low	Low
Vibrations	Medium	High	Medium	High	Medium
